# Beyond expression: structured art-based therapy and attention span in pupils with intellectual disability

**DOI:** 10.3389/frcha.2026.1798631

**Published:** 2026-06-03

**Authors:** Udeme Samuel Jacob, Sri Nurhayati, Mbulaheni Obert Maguvhe

**Affiliations:** 1 Postdoctoral Research Fellow, Department of Inclusive Education, School of Educational Studies, College of Education, University of South Africa, Pretoria, South Africa; 2 Department of Community Education, Postgraduate Program, Institut Keguruan dan Ilmu Pendidikan Siliwangi, Cimahi, Indonesia; 3 Department of Inclusive Education, School of Educational Studies, College of Education, University of South Africa, Pretoria, South Africa

**Keywords:** art therapy, attention span, cognitive intervention, degree of disability, inclusive education, intellectual disability

## Abstract

**Introduction:**

Attentional deficits remain a significant barrier to learning among pupils with intellectual disability, particularly in resource-constrained educational settings where access to specialised interventions is limited. This study examined the effect of a structured art-based intervention on attention span and explored the moderating role of disability severity among pupils with intellectual disability.

**Methods:**

A quasi-experimental pre-test-post-test control group design with a 2 × 2 factorial structure was employed. Twenty pupils with mild and moderate intellectual disability were purposively selected and assigned at the school level to intervention and control groups. The intervention consisted of 24 structured sessions integrating painting, clay modelling, music, and guided reflection activities designed to promote sustained attentional engagement. Attention span was measured using the Moss Attention Rating Scale, while data were analysed using analysis of covariance.

**Results:**

Results revealed a statistically significant improvement in attention span among pupils exposed to the intervention, with a large effect size. Pupils with mild intellectual disability demonstrated higher post-test attention scores than those with moderate disability, although the interaction effect was not statistically significant.

**Discussion:**

The findings suggest that structured art-based intervention may enhance attentional engagement among pupils with intellectual disability and provide a low-cost, teacher-implemented strategy for inclusive classrooms in low-resource contexts. However, findings should be interpreted cautiously due to the small sample size and cluster-based design.

## Introduction

Attention span constitutes a fundamental cognitive resource that underpins learning, memory consolidation, and classroom participation, yet it is often compromised in pupils with intellectual disabilities (ID). These deficits are not merely secondary features but rather represent core barriers to effective education, manifesting as difficulties in sustaining focus, following instructions, and completing tasks (1, 2). As emphasized in multimedia-learning theory, visual and verbal processing jointly support comprehension and memory (3), highlighting why attention lapses critically undermine classroom learning. Children with ID frequently exhibit behaviors comparable to those without ID but with greater persistence and intensity (4). Such challenges directly interfere with academic performance and social integration, limiting the educational trajectories of these pupils. Because attentional control is shaped by neurocognitive, behavioral, and contextual factors, recent authors advocate integrated, multidimensional interventions that address these domains simultaneously (5, 6).

Research consistently highlights that pupils with moderate intellectual disabilities (MID) face especially acute attentional deficits, including a markedly short attention span, impulsivity, and difficulty regulating activity levels (1, 4). These attention-related behaviors disrupt instructional flow and peer interaction, echoing findings that sustained attention is pivotal for adaptive classroom behavior (7). At the cognitive level, deficits in visual attention have been reported among children with Down syndrome (2). Such weaknesses reduce the pedagogical value of visual aids and multimodal materials (3). Behavioral attention difficulties measured by parents and teachers correlate strongly with poor literacy and numeracy outcomes (2). Collectively, this evidence underscores the functional impact of attentional deficits and the need for structured and contextually relevant intervention approaches.

The research problem becomes sharper when considering the intersection of cognitive deficits, behavioral patterns, and environmental contexts. Intellectual and developmental disabilities (IDD) are consistently associated with heightened attentional difficulties (8). Studies show that children with ID struggle with selective attention tasks (9). Environmental attributes also exert measurable influence (10). Recent art-therapy literature frames these interactions through the Expressive Therapies Continuum, which posits that sensory, perceptual, and cognitive responses co-shape learning (11, 12). Thus, attention span deficits must be understood as products of dynamic interactions between cognitive functioning and environmental conditions.

Despite growing recognition of these challenges, the dominant responses remain fragmented. Pharmacological and behavioral approaches carry limitations (13, 14). Recent authors note that art-based approaches offer a cost-effective, non-pharmacological alternative for sustaining attention and engagement (5, 15). Hence, structured creative interventions are increasingly promoted as context-sensitive solutions in low-resource educational settings, where access to specialised services remains limited (6). Converging school-based studies indicate that art-based programs can be embedded into classroom routines and are associated with improvements in classroom engagement and socio-emotional functioning (16–18). These findings underscore the feasibility of integrating structured art-based interventions into everyday classroom practice, particularly in contexts where low-cost, teacher-delivered strategies are required.

Against this backdrop, scholars have tested a range of non-pharmacological approaches. These approaches align with findings that multimodal and sensory-driven activities stimulate neural networks supporting attention and executive control (11). Music therapy and pictorial illustration have shown promise in enhancing attention, especially in children with mild ID (19). Therapeutic play has also demonstrated efficacy in improving focus and engagement (20), while digital innovations like the Sondam Rhythm Game have improved attention through gesture-based learning (21). Similarly, computerised cognitive training has produced gains in selective attention, although sustained attention benefits are inconsistent (8). These approaches converge on the principle that multimodal, interactive, and play-based strategies engage attentional processes more effectively than traditional didactic methods.

Among these strategies, structured art-based interventions have emerged as particularly promising. Such interventions involve guided, goal-oriented creative activities to support cognitive, motor, and emotional development rather than solely expressive outcomes (11, 12). In this study, the term structured art-based intervention is used to distinguish the programme from clinical art therapy, as it was implemented by trained educators rather than licensed art therapists and focused on structured cognitive and instructional outcomes. When systematically implemented, structured art-based interventions have been shown to improve attention, task persistence, and learning behaviours (6, 7). By supporting emotional well-being and behavioral adjustment, structured art-based intervention indirectly strengthens pupils’ capacity for sustained focus (22, 23). Global trends further reinforce this potential. Recent scoping reviews highlight robust evidence for art-based interventions enhancing attention and executive function across ages (15, 24). The severity of disability has been shown to shape responsiveness (9, 25). These findings underscore the importance of testing interaction effects between therapy type and disability severity using factorial designs.

In the African context, and specifically in Nigeria, research on structured art-based interventions in educational settings remains limited. Systemic barriers restrict integration (26, 27). Empirical studies are rare (28). However, recent African research confirms that art-based approaches enhance non-verbal communication and cultural engagement (24). Despite limited formal training in Nigeria, school-based models led by teachers and community artists are emerging as viable alternatives (15). This highlights a critical need for contextually grounded, school-based research that evaluates both feasibility and effectiveness within low-resource environments. Addressing these gaps is both academically and socially urgent (29, 30).

This study was therefore designed to address the dual gap in both empirical evidence and contextual application. Specifically, it sought to evaluate the effect of a structured art-based intervention on attention span in pupils with mild and moderate ID in Nigeria, using a quasi-experimental, pre-test-post-test control-group design with a 2 × 2 factorial structure. The research tested three null hypotheses: that there would be no significant main effect of structured art-based therapy, no significant main effect of degree of disability, and no significant interaction effect of structured art-based therapy and disability level on attention span outcomes. By adapting the Moss Attention Rating Scale (MARS) to this context, the study also contributes psychometric innovation, extending the instrument's use beyond its traditional application in traumatic brain injury research (19).

The novelty of this research lies in four dimensions. First, it reconceptualises structured art-based intervention as a cognitive and instructional tool rather than an expressive add-on. Second, it provides one of the first controlled empirical investigations within the Nigerian context. Third, it introduces a factorial design that tests not only treatment effects but also the moderating role of disability severity. Fourth, it validates the MARS scale in intellectual disability populations, broadening its scope of application. Within the scope of 20 purposively sampled pupils from special schools in Ibadan, this study contributes to global debates on non-pharmacological interventions for ID while offering locally relevant insights for Sub-Saharan Africa. In doing so, it addresses the urgent need for scalable, evidence-based, and context-sensitive strategies to enhance educational participation and outcomes for children with intellectual disabilities. The objectives of the study are:

1. To examine the effect of a structured art-based intervention on attention span among pupils with intellectual disability
2. To determine the effect of the degree of disability on attention span outcomes
3. To investigate the interaction effect between intervention and degree of disability on attention span

## Literature review

### Theoretical foundations of attention development in learners with intellectual disability

Understanding the mechanisms that shape attention span in pupils with intellectual disability requires grounding in theories of cognitive development, executive functioning, and the role of play in learning. Foundational frameworks from Lev Vygotsky, a Soviet developmental psychologist, and Jean Piaget, a Swiss cognitive theorist, emphasize that learning occurs through active engagement and scaffolding within social contexts, with play serving as a developmental medium for cognitive and self-regulatory growth. Vygotsky's concept of the “zone of proximal development” highlights guided play as a means of extending attentional reach, while Piaget viewed play as a process of symbolic assimilation and schema consolidation that demands focused attention and reflection. Recent neurocognitive theories, such as the Expressive Therapies Continuum (11, 12), extend these foundations by framing art-based activity as a hierarchical process activating sensory, perceptual, affective, and cognitive levels of response. These frameworks align with evidence that attention span functions as a cornerstone for inhibitory control, working memory, and cognitive flexibility (8, 9). Theories of neural plasticity further explain how interventions that engage multiple sensory modalities, such as art or play-based approaches, can strengthen neural networks for sustained focus and self-regulation (31, 32). Thus, art therapy can be conceptualized not only as expressive practice but as a neurodevelopmentally grounded intervention for enhancing attention.

### Conceptualisation of structured art-based intervention

A structured art-based intervention refers to a standardised, activity-focused, and facilitator-guided programme that uses planned creative tasks to achieve defined cognitive, behavioural, psychosocial, or functional outcomes (33–35). It is characterised by deliberate organisation of content, sequencing, and delivery, ensuring that activities are systematically designed to produce measurable effects (36, 37). In contrast to unstructured creative engagement, such interventions emphasise consistency, replicability, and goal-directed implementation.

Operationally, four indicators define these interventions. First, they involve predefined activities and materials, such as drawing, painting, collage, clay, or craft-based tasks, aligned with specific objectives (33, 38). Second, they follow a clear session structure, typically comprising orientation, guided activity, practice, reflection, and closure, delivered in a consistent sequence, which supports engagement and instructional flow (35, 36). Third, they maintain a fixed intervention dose, defined by the number, duration, and frequency of sessions, enhancing standardisation and comparability across contexts (34, 39). Fourth, they incorporate training and fidelity procedures, including facilitator preparation and adherence monitoring, to ensure alignment with the intended protocol, thereby strengthening implementation consistency (37, 40).

It is essential to distinguish structured art-based interventions from clinical art therapy, as the two differ fundamentally in design and implementation. Clinical art therapy is a psychotherapeutic, therapist-led intervention grounded in formal psychological frameworks and aimed at treating mental health conditions or complex emotional difficulties (41, 42). Its implementation is guided by individualised case formulation and flexible adaptation, with emphasis on processes such as emotion regulation, interpersonal dynamics, and self-expression (39).

In contrast, structured art-based interventions are non-psychotherapeutic, protocol-driven programmes delivered by teachers, instructors, or trained facilitators rather than licensed art therapists. Their primary aim is to enhance cognitive engagement, participation, and functional outcomes rather than provide psychotherapy (33, 38, 43). Consequently, the distinction lies in purpose, facilitator expertise, therapeutic intent, and implementation logic: structured interventions prioritise standardisation and measurable outcomes, whereas clinical art therapy prioritises therapeutic responsiveness and psychological change processes (35, 41).

### Attention span difficulties in pupils with intellectual disability

Empirical research confirms that pupils with intellectual disabilities (ID) face pronounced attentional difficulties across classroom and everyday contexts (1, 2). These behaviors—distractibility, impulsivity, and hyperactivity—occur with greater persistence and intensity (4). Such manifestations reflect a breakdown in both top-down (executive) and bottom-up (sensory) attention processes, consistent with the dual-attention model proposed by Hinz (11) in expressive therapies research. Short attention span is particularly evident in pupils with moderate intellectual disabilities, limiting classroom engagement. Cognitive deficits, including poor visual vigilance and inefficient search strategies, reduce responsiveness to instructional cues (2). Recent evidence shows that attention impairments in ID populations also correlate with emotional dysregulation, suggesting that interventions combining sensory engagement and emotional expression, such as structured art-based therapy, can improve focus and behavioral self-regulation (5, 6). Together, these findings underscore the multidimensional nature of attentional difficulties in ID and the need for multimodal intervention models.

### Degree of disability and responsiveness to intervention

The degree of intellectual disability further influences attentional performance and responsiveness to intervention. Mild intellectual disability (MID) is typically associated with IQ scores between 55 and 69, whereas moderate disability falls between 35 and 55 (61, 62). Research demonstrates that children with mild ID retain stronger selective attention and working memory, allowing greater responsiveness to targeted interventions (44). Art-therapy studies show similar patterns: pupils with mild ID exhibit stronger engagement and creative fluency, while those with moderate ID benefit from simplified, sensory-level activities (6, 7). Structured physical activities (63) and behavioral programs (45) demonstrate differential gains by severity, reinforcing the need for stratified approaches. Integrating expressive and motor-based components, as seen in structured art-based therapy, can bridge this gap by supporting both cognitive and affective regulation across levels of impairment (11).

### Existing interventions for attention span

Several intervention models have been employed to improve attention span among children with ID, spanning computerized, behavioral, physical, and classroom-based approaches. However, few integrate creative and cognitive dimensions in a sustained format. Art-based interventions uniquely combine sensory engagement, executive activation, and emotional expression, forming a bridge between traditional behavioral and cognitive training (5, 15). These non-pharmacological approaches are also markedly more cost-effective than pharmacological treatments, which often require ongoing medical supervision, imported medication, and specialized personnel. In contrast, structured art-based therapy utilizes locally available materials and trained facilitators, making it a scalable and sustainable option for inclusive education in low-resource contexts. This growing recognition underscores that attention can be strengthened through multimodal engagement rather than rote repetition.

### Art-based therapies and attention outcomes

Within this landscape, art-based interventions occupy a distinctive position by combining cognitive, emotional, and sensory mechanisms. Structured art-based therapy sessions have demonstrated positive effects on attention span and broader cognitive performance (64). From a neurobiological standpoint, structured art-based therapy activates visual–motor integration pathways and stimulates prefrontal regions associated with attentional control (12, 31). Meta-analyses confirm small to medium cognitive gains (65). Emerging evidence also supports its educational applicability, with structured art-based programs improving engagement and task completion in pupils with developmental disorders (6, 15). Thus, art therapy functions as a structured, evidence-informed strategy aligning with multimodal learning theories.

Nevertheless, critical gaps remain in the literature. Despite these benefits, relatively few controlled studies have examined its impact on children with intellectual disability, particularly in low-resource settings. Research that does exist often fails to disaggregate outcomes by severity of disability, leaving open whether pupils with mild ID benefit differently from those with moderate ID. Furthermore, in many African contexts, including Nigeria, the absence of institutionalized art therapy programs and limited policy frameworks constrain both research and practice (26, 30). Addressing these challenges requires studies that are both methodologically rigorous and culturally adapted.

Synthesizing across theoretical, empirical, and applied perspectives, the justification for the present study becomes evident. Attention span deficits are a defining challenge for pupils with intellectual disability, with implications for academic achievement, social integration, and emotional well-being. While multiple interventions have been trialed, art therapy offers a uniquely multimodal pathway that engages cognitive, sensory, and emotional processes simultaneously. Evidence supports its potential across age groups and conditions, yet robust, context-sensitive studies in Sub-Saharan Africa are absent. Furthermore, few investigations have examined the moderating role of degree of disability in shaping responsiveness to art therapy, leaving a critical blind spot in both research and practice. By employing a quasi-experimental factorial design and adapting the Moss Attention Rating Scale (MARS) for ID populations, the current study aims to generate the first controlled evidence from Nigeria on the cognitive benefits of art therapy. This addresses not only a scholarly gap but also a practical need for scalable, low-cost, non-pharmacological interventions within inclusive education systems. While the number of professionally trained art therapists in Nigeria remains limited, emerging teacher-facilitated and community-artist models offer feasible alternatives for implementing structured art-based therapy in schools (27). In reframing structured art-based therapy from enrichment to evidence-based cognitive intervention, the study advances theoretical debates on attention and executive function, contributes empirical knowledge on severity-specific responsiveness, and offers policy-relevant insights for special education in resource-limited contexts.

## Method

### Research design

A quasi-experimental 2 × 2 pre-test post-test control design was employed, as previously outlined, to examine both main and interaction effects (46). Quasi-experimental designs are widely employed in special education intervention research because they permit causal inference in authentic educational settings where full randomization is impractical (46). The factorial format enabled simultaneous examination of two independent factors: treatment condition (structured art-based therapy vs. control) and degree of disability (mild vs. moderate). This structure was selected for its efficiency, as factorial designs allow the evaluation of main and interaction effects while maximizing information gained from relatively small samples—a common challenge in disability studies (47). While quasi-experimental designs face challenges such as assumptions of group equivalence and access to adequate controls (48). They remain a best-practice choice for balancing methodological rigour with ecological validity in special education research. This design thus provided a suitable framework to test whether structured art-based therapy enhanced attention span and whether responsiveness varied by disability severity.

In addition, randomisation was implemented at the school level (cluster randomisation) rather than at the individual level to preserve intact classroom structures and minimise contamination between participants. Schools, therefore, constituted the unit of allocation, while pupils remained the unit of analysis.

### Participants and sampling strategy

The sample consisted of 20 pupils with intellectual disability recruited from two special schools in Ibadan, Oyo State, Nigeria. Inclusion criteria were classification as mild or moderate intellectual disability, confirmed by psychological assessment. Purposeful sampling was used to ensure participants reflected the study's target population, a strategy recommended in disability research to capture relevant variation (49). Following selection, participants were randomly assigned to either the experimental group (*n* = 10) or control group (*n* = 10).

Random allocation was conducted at the school level using a simple randomisation procedure (e.g., computer-generated random numbers), with assignment performed by the research team prior to intervention delivery. Allocation was not influenced by participant characteristics, thereby reducing selection bias.

To avoid contamination of treatments, groups were drawn from different schools. This approach reflects a cluster allocation design commonly adopted in school-based interventions, where physical and instructional proximity may otherwise compromise treatment integrity. The experimental group (mean age = 16.2 years, SD = 3.55) received the intervention, while the control group (mean age = 14.8 years, SD = 4.39) followed standard routines without additional support. Random allocation enhanced internal validity, while school-level separation strengthened control over confounding variables. Allocation was conducted prior to intervention delivery and was not influenced by participant characteristics. Recruitment procedures adhered to best practices identified in research with children with disabilities: direct engagement with schools and families ensured high participation while minimizing attrition.

### Intervention: structured art-based intervention protocol

The intervention comprised 24 structured sessions delivered over 12 weeks (2 per week), each lasting 45 min. The programme was designed to promote multisensory engagement, sustain attention, and behavioural regulation through a structured and progressively sequenced approach. Each session followed a standardised format consisting of (1) a structured greeting song (5 min), (2) guided art activity (25 min), (3) independent or supported practice (10 min), and (4) reflection and closing song (5 min).

Across the intervention period, activities were organised thematically and developmentally to ensure progression in cognitive and motor demands. Early sessions (Weeks 1–3) emphasised sensory exploration and gross motor engagement through activities such as free painting, large brush strokes, and broad hand movements to capture attention and build initial engagement. Middle sessions (Weeks 4–8) introduced more structured tasks, including guided drawing, collage-making, and shape formation, which required increased focus, sequencing, and task completion. Later sessions (Weeks 9–12) focused on fine motor precision, detailing, tracing, and independent task execution, alongside more structured reflection and peer interaction. This progression was designed to scaffold attentional control from simple to complex task demands.

Each session began with a structured greeting song to orient pupils and establish routine, followed by creative tasks such as painting, clay modelling, collage-making, and themed projects (e.g., nature and animals). Background calming music was incorporated in selected sessions to support focus and regulate arousal levels. Reflection activities were systematically embedded at the end of each session, during which pupils described their work, responded to prompts (e.g., “What did you create?” and “What part was most interesting?”), received feedback, and practised turn-taking and communication skills.

Instruction was delivered by a trained facilitator and supported by a research assistant. The facilitator, who held a master's degree in special education and had prior experience in art-based pedagogy, led all instructional activities and guided task execution. The facilitator received 1 week of structured training on the intervention protocol, including session delivery, activity sequencing, learner engagement strategies, and behavioural support, under the supervision of an art therapy consultant. The research assistant was responsible for behavioural observation, fidelity monitoring, and session documentation.

To ensure fidelity of implementation, a structured checklist was used to monitor adherence to session objectives, activity sequence, instructional procedures, appropriate use of materials, learner engagement, and completion of reflection activities. Fidelity observations were conducted by the research team throughout the intervention, with an overall adherence rate of approximately 90%, indicating high consistency in implementation across sessions.

Safety considerations were integrated into the intervention. Participants who exhibited signs of distress, fatigue, or behavioural difficulty during sessions received immediate support from the facilitator. Where necessary, pupils were referred to appropriate school-based support services in accordance with ethical guidelines. All activities were adapted to match pupils' developmental levels, ensuring accessibility and minimising risk.

The programme is described as a structured art-based intervention rather than formal art therapy, as it was implemented by trained educators under professional guidance rather than licensed art therapists. Oversight was provided through consultation with an art therapy specialist to ensure adherence to appropriate principles and practices.

This protocol integrates motor, emotional, and cognitive processes within a structured instructional framework, aligning with evidence that art-based, play-oriented activities support sustained attention, engagement, and socio-emotional development (6).

### Instrumentation

Attention span was measured using the Moss Attention Rating Scale (MARS), a 22-item tool initially developed for assessing attention following traumatic brain injury (66). Alternative measures such as the Scale of Attention in Intellectual Disability (SAID) or the Continuous Performance Test (CPT) were considered; however, MARS was selected for its strong ecological validity, direct behavioral observation format, and prior adaptation for children with intellectual disabilities in educational settings (19). Items cover distractibility, initiation, sustained focus, and hyperactivity, scored on a five-point Likert scale and convertible to a standardized 0–100 scale. The MARS has demonstrated ecological validity and inter-rater reliability, with subsequent studies reporting acceptable internal consistency among individuals with moderate intellectual disability (19). For this study, administration procedures were adapted for Nigerian classrooms, with raters trained to ensure cultural and developmental appropriateness.

The adaptation process included modifying administrative procedures to align with classroom practices, as well as rater training and calibration to ensure scoring consistency. Internal consistency and inter-rater reliability were evaluated for the present sample to support measurement validity. Score direction was standardised such that higher values indicated improved attention performance.

### Data collection procedure

Baseline (pre-test) attention scores were collected using the MARS before the intervention began. The experimental group then received art therapy, while the control group followed their usual classroom activities. After 12 weeks, post-test scores were obtained using the same instrument and raters. Raters were blinded to group assignments to minimize bias. Observations and scoring followed strict guidelines, consistent with recommendations for ensuring reliability in disability research (50). Scoring procedures were standardised across raters, including the application of the 0–100 transformation protocol, to ensure comparability of pre- and post-test measures.

### Data analysis

Analysis of covariance (ANCOVA) was conducted to test the effect of art therapy on post-test scores while controlling baseline differences. ANCOVA was chosen for its ability to adjust for covariates and strengthen causal inference in quasi-experimental studies (51).

Given the cluster-based allocation of participants, analyses were interpreted with consideration of potential intra-cluster dependence. Where appropriate, statistical adjustments (e.g., robust standard errors) were applied to account for clustering at the school level. In addition, key ANCOVA assumptions, including homogeneity of regression slopes (Group × Pretest interaction), normality of residuals, and homoscedasticity, were examined prior to interpretation of results. Homogeneity of regression slopes was satisfied as the interaction between group and pre-test scores was not significant (*p* > .05). Visual inspection of standardised residuals and normal probability plots indicated no serious deviation from normality. Homoscedasticity was also supported by the distribution of residuals across predicted values.

Bonferroni-adjusted *post hoc* tests were applied to compare group means. Pairwise comparisons were further examined using estimated marginal means, with corresponding mean differences, 95% confidence intervals, and adjusted *p*-values reported to enhance interpretability. Standardised effect sizes were also computed to quantify the magnitude of observed effects. Effect sizes were estimated using partial eta squared (*η*^2^) to quantify treatment magnitude. All analyses were conducted with SPSS statistical software.

### Validity and reliability

Several measures ensured the study's rigor. Internal validity was enhanced through random assignment, separation of groups across schools, and statistical control of pre-test scores. External validity was supported by selecting participants from multiple schools, increasing generalizability within the Nigerian context. Construct validity was supported using the Moss Attention Rating Scale (MARS), which has been previously validated for assessing observable attentional behaviours, including sustained attention, distractibility, and initiation, in populations with cognitive impairments. Reliability was strengthened by training raters, conducting inter-rater checks, and blinding them to treatment condition. Raters were trained by the principal investigator, who is a certified educational psychologist, through two calibration sessions using sample video recordings to standardize MARS scoring procedures. These steps minimized observer bias and maximized consistency. Cultural responsiveness was operationalized by integrating local materials, familiar themes, and bilingual instructions (English and Yoruba), as well as classroom songs and motifs drawn from Nigerian cultural settings, ensuring that pupils' participation reflected their everyday learning experiences, following recommendations for context-sensitive intervention research (52).

However, given the small number of clusters (two schools), the precision of cluster-level estimates is limited. Findings should therefore be interpreted with caution, particularly with respect to generalisability and statistical power.

### Ethical considerations

Parental or guardian informed consent was obtained prior to participation, and assent was sought from pupils using age-appropriate explanations. Consent was treated as an ongoing process, allowing participants the right to withdraw at any stage without consequence (53). Confidentiality was maintained by anonymizing data and securing records.

The study adhered to established ethical guidelines for research involving minors, with particular attention to safeguarding participant welfare. In line with these standards, de-identified data are available from the corresponding author upon reasonable request, rather than through public data repositories. Respect for autonomy and dignity remained central throughout the study, consistent with calls to promote the equal worth and active inclusion of individuals with intellectual disabilities in research (54). Interventions were integrated into school routines to minimise disruption and ensure pupil well-being.

## Results and discussion

This section presents findings directly addressing the study's purpose: to determine whether structured art-based therapy enhances attention span in pupils with intellectual disability and whether responsiveness varies by disability severity. The section also connects these outcomes to the study's hypotheses and to its broader theoretical goal of identifying scalable, non-pharmacological strategies for cognitive development. Results are organized according to these hypotheses and include statistical outcomes and their practical implications.

### Analysis of covariance

To test the study hypotheses, an Analysis of Covariance (ANCOVA) was conducted, controlling for pre-test attention span scores. Before conducting ANCOVA, the key assumptions were tested. The homogeneity of regression slopes assumption was satisfied, as the interaction between group and pre-test scores was not statistically significant (*p* > .05). Residual diagnostics further indicated approximate normality and homoscedasticity. Given that randomisation was implemented at the school level, results were interpreted with consideration of potential intra-cluster dependence, and robust standard errors were applied where appropriate. The outcomes are summarised in Tables 1–3.

**Table 1:** Analysis of covariance of post-attention span of pupils with intellectual disability by treatment and degree of disability.

Source	Type III sum of squares	Df	Mean square	*F*	Sig.	Partial eta squared
Corrected model	1,256.198^a^	4	314.050	4.823	.011	.563
Intercept	1,175.785	1	1,175.785	18.057	.001	.546
Pretest score	86.665	1	86.665	1.331	.267	.081
Treatment	732.086	1	732.086	11.243	.004	.428
Degree of disability	299.503	1	299.503	4.599	.049	.235
Treatment × degree of disability	35.381	1	35.381	.543	.472^b^	.035
Error	976.752	15	65.117			
Total	74,113.000	20				
Corrected total	2,232.950	19				

a *R* squared = .563 (adjusted *R* squared = .446).

b All reported effects reflect adjusted outcomes controlling for baseline attention scores.

**Table 2:** Estimated marginal means of post-attention span by treatment.

Treatment	Mean	Std. error	95% CI lower	95% CI upper
Art therapy	65.551	2.686	59.825	71.277
Control group	52.686	2.637	47.065	58.308

Adjusted mean difference = 12.865. Approximate Hedges’ *g* = 1.53, 95% CI [0.55, 2.50]. Adjusted for the covariate: pre-test attention span = 47.40.

**Table 3:** Bonferroni *post hoc* analysis of attention span by degree of disability.

(I) Degree of disability	(J) Degree of disability	Mean difference (I-J)	Std. error	Sig.	95% CI lower	95% CI upper
Mild	Moderate	8.314*	3.877	.049	.051	16.576
Moderate	Mild	−8.314*	3.877	.049	−16.576	−.051

* Significant at *p* < .05, Bonferroni adjusted.

Table 4 presents baseline characteristics of participants in the art therapy and control groups. No statistically significant differences were observed across variables (*p* > .05), indicating general baseline comparability. The art therapy group was slightly older (*M* = 16.20, SD = 3.55) than the control group (*M* = 14.80, SD = 4.39), *t*_(18)_ = 0.79, *p* = .441, while sex distribution was similar, *χ*^2^ = 0.40, *p* = .527. A higher proportion of mild disability was observed in the art therapy group (70%) than in the control group (40%), though the difference was not statistically significant (*χ*^2^ = 1.67, *p* = .197), supporting its inclusion as a covariate. Baseline MARS scores and comorbidity status were also comparable (*p* > .05). Given cluster-based allocation and small sample size, findings should be interpreted cautiously.

**Table 4:** Baseline characteristics of participants by treatment group.

Variable	Art therapy (*n* = 10)	Control (*n* = 10)	Test statistic	*p*-value
Age (years), mean (SD)	16.20 (3.55)	14.80 (4.39)	*t* = 0.79	.441
Sex, *n* (%)		*χ*^2^ = 0.40	.527	
Male	6 (60%)	5 (50%)		
Female	4 (40%)	5 (50%)		
Degree of disability, *n* (%)		*χ*^2^ = 1.67	.197	
Mild	7 (70%)	4 (40%)		
Moderate	3 (30%)	6 (60%)		
Baseline MARS score, mean (SD)	48.10 (6.25)	46.70 (5.90)	*t* = 0.52	.612
Comorbidity/medication, *n* (%)			*χ*^2^ = 0.22	.639
Yes	2 (20%)	3 (30%)		
No	8 (80%)	7 (70%)		

### Tests of between-subjects effects

#### Dependent variable: post-test (attention span)

Table 1 shows a statistically significant main effect of treatment on post-test attention span after controlling for pretest scores and degree of disability, *F*_(1, 15)_ = 11.243, *p* = .004, partial *η*^2^ = .428. This indicates that the structured art-based intervention produced a substantial improvement in attention span. The adjusted mean difference between the intervention and control groups was 12.865 points. The corresponding approximate standardized effect was large, Hedges' *g* = 1.53, 95% CI [0.55, 2.50]. The degree of disability also had a significant effect, *F*_(1, 15)_ = 4.599, *p* = .049, partial *η*^2^ = .235. Pupils with mild disability scored higher than those with moderate disability, with an adjusted mean difference of 8.314 points, corresponding to an approximate Hedges' *g* = 0.99, 95% CI [0.09, 1.89]. The interaction between treatment and degree of disability was not significant, *F*_(1, 15)_ = 0.543, *p* = .472, partial *η*^2^ = .035, suggesting no statistically reliable evidence of differential effects across disability levels; however, this finding should be interpreted cautiously given the limited statistical power. All reported effects reflect adjusted outcomes controlling for baseline attention scores.

Table 2 presents the estimated marginal means adjusted for pre-test scores. The intervention group achieved a higher adjusted mean (*M* = 65.55, SE = 2.69) compared to the control group (*M* = 52.69, SE = 2.64), with an adjusted mean difference of 12.865 points. This difference represents a large standardized effect (Hedges' *g* = 1.53, 95% CI [0.55, 2.50).

The effect size for disability level (*η*^2^ = .235) was moderate, indicating that the severity of intellectual disability is an important predictor of attentional outcomes. Pupils with milder impairments responded more strongly to the intervention. Bonferroni-adjusted pairwise comparisons confirmed that this difference was statistically significant (mean difference = 8.314, 95% CI [0.051, 16.576], *p* = .049), supporting the robustness of the observed group differences.

The first major finding confirmed that structured art-based intervention significantly enhanced the attention spans of pupils with intellectual disabilities. After controlling for pretest scores and degree of disability, the intervention group demonstrated a large effect size (partial *η*^2^ = .428), explaining 42.8% of post-test variance. This magnitude indicates that pupils in the intervention group demonstrated substantially improved ability to sustain attention during classroom tasks, with direct implications for task completion, instructional engagement, and learning readiness. This underscores that structured art-based intervention, defined here as a sequenced, facilitator-guided model emphasising graded sensory-to-cognitive task functions, operates as a robust cognitive intervention rather than an expressive enrichment activity.

The interaction between treatment and disability severity was not significant [*F*_(1, 15)_ = 0.543, *p* = .472, *η*^2^ = .035]. While pupils with mild disabilities generally outperformed those with moderate disabilities, both groups benefited similarly from art therapy. The absence of an interaction effect suggests that the intervention's impact was consistent across severity categories. However, given the relatively small sample size (*n* = 20) and the cluster-based allocation of participants, this non-significant interaction should be interpreted cautiously, as the study may have been underpowered to detect subtle interaction effects.

This consistency may also reflect sample variability in age and cognitive profiles, which could have reduced statistical power to detect subtle differences. Nevertheless, the result reinforces the adaptability of art therapy for heterogeneous classrooms.

Sensitivity analysis considering the clustered structure of the data indicated that the pattern and significance of results remained stable after adjustment, suggesting that the observed treatment effect is robust. However, the small number of clusters (two schools) limits the precision of cluster-level estimates and warrants cautious interpretation.

## Discussion

These findings extend existing literature by providing the first controlled evidence from a Nigerian school context that art therapy enhances attention span in pupils with intellectual disability. Previous research has primarily examined emotional or behavioral outcomes or involved neurotypical or adult populations. The present study advances this field by demonstrating comparable cognitive gains among pupils with ID, suggesting that the mechanisms identified internationally, such as neural plasticity and sustained engagement (31, 32), are also active in low-resource educational contexts.

The robustness of the treatment effect is noteworthy given the modest sample size. Bonferroni-adjusted comparisons showed a 12.87-point gain for the art therapy group, indicating strong practical significance. This study advances the field by positioning structured art-based therapy as a neurocognitively grounded, classroom-feasible intervention. Prior studies have emphasized emotional or behavioral outcomes (55), but this research demonstrates that attention, a foundational cognitive process underpinning learning, can be systematically strengthened through guided creative engagement, supporting its integration in school-based special education practice.

Alternative explanations, such as placebo effects or increased teacher attention, are unlikely, as both groups received equivalent levels of instructor engagement and supervision. The structured 12-week protocol, delivered consistently across sessions, and the non-overlapping confidence intervals between groups further support the conclusion that the observed gains were due to the art therapy intervention rather than external factors.

A second major finding was the significant effect of disability severity on post-test attention outcomes. Pupils with mild intellectual disability achieved higher adjusted scores than those with moderate disability. The effect size (partial *η*^2^ = .235) indicated a moderate impact, highlighting disability severity as a meaningful predictor of responsiveness. This finding aligns with international evidence that baseline cognitive capacity influences responsiveness to intervention. Children with mild ID often show greater cognitive flexibility, enabling stronger responses to structured programs (44). In contrast, individuals with moderate ID may require longer, more scaffolded supports (45, 56). The present study adds context-specific insight by confirming that even with simplified, culturally adapted tasks, art therapy benefits both groups—extending global findings to African special education contexts.

These findings add nuance to previous research: while both groups benefited, pupils with mild disability demonstrated greater gains, likely due to stronger baseline working memory and attentional control. Nevertheless, improvements in the moderate group—though smaller—confirm that art therapy remains effective across severity levels. This supports its use in inclusive classrooms and underscores the need for differentiated protocols (e.g., simplified steps, visual cues, extended time) to ensure accessibility for learners with moderate disabilities.

Contrary to expectations, the interaction between treatment and disability severity was not significant, indicating similar improvement patterns across mild and moderate groups. While mild disability pupils achieved higher absolute scores, the proportional benefits were comparable. The absence of an interaction may reflect both limited statistical power due to small sample size and developmental variability (age and SD differences). Nonetheless, the result underscores a major strength of art therapy—its adaptability across cognitive and functional levels. Because it integrates multisensory, emotional, and non-verbal components, it remains accessible even for pupils with greater impairment, supporting its scalability as a universal classroom strategy.

Taken together, the findings provide novel evidence that structured art-based therapy—defined as a sequenced, facilitator-guided model emphasizing progressive sensory-to-cognitive tasks—significantly enhances attention span among pupils with intellectual disability. Stronger outcomes were observed for mild disability, though benefits extended across severity levels. This study's distinct contribution lies in demonstrating, through a quasi-experimental factorial design, that art therapy functions as a cognitive intervention, comparable in efficacy to cognitive training and play-based models, rather than solely as an expressive outlet.

These findings translate directly into classroom practice by providing teachers with a structured, low-cost framework for improving attention in learners with intellectual disability. For classroom practice, the findings suggest that structured art-based intervention can be integrated into daily teaching routines using low-cost materials such as paper, paint, and clay. Teachers can implement short, structured sessions that follow a predictable sequence of guided activity, practice, and reflection to enhance pupils' attentional engagement. The progression from simple motor activities to more detailed tasks provides a practical framework for scaffolding attention in learners with varying levels of intellectual disability. In resource-constrained settings, this approach offers a feasible alternative to specialist-led interventions, enabling teachers to support attention development within inclusive classrooms. Policy-level recognition of art therapy as an allied educational support, along with integration into teacher preparation curricula, would enhance its reach and sustainability (57, 58).

The quasi-experimental design and cluster-based allocation introduce potential sources of bias, including residual confounding and intra-cluster dependence. Although statistical adjustments were applied, these factors may influence the precision and generalisability of the estimates. Despite its strengths, the study is not without limitations. The small sample size and single-site context limit generalizability, and the short intervention period prevents conclusions about long-term sustainability of gains. The lack of stratified analyses within the moderate disability group also restricts insights into intra-group variability. Furthermore, although the Moss Attention Rating Scale provided reliable measures, reliance on a single assessment tool may not fully capture the multidimensionality of attention. The study did not assess the long-term sustainability of attention gains beyond the intervention period. It remains unclear whether observed improvements would be maintained over time without continued structured support.

Future research should address these limitations through multi-site randomized trials with larger and more diverse samples, extended follow-up periods, and multimodal measurement approaches combining behavioral, neurocognitive, and neurobiological indicators. Cross-cultural comparisons would also be valuable to determine how contextual factors shape outcomes, particularly in underrepresented regions such as Sub-Saharan Africa, where art therapy research remains sparse (26, 27).

This study suggests that structured art-based intervention may serve as an effective and scalable approach that enhances attention span among pupils with intellectual disability. The intervention yielded substantial improvements, especially for mild disability and consistent benefits across severity levels. These results expand educational art therapy research and highlight actionable applications in inclusive, low-resource contexts. By reframing art therapy as a cognitive, not merely expressive, intervention, the study offers evidence with clear implications for both practice and policy. Due to the small sample size, multilevel modelling was not feasible, limiting the ability to explicitly account for clustering effects and potentially affecting the precision of the estimates.

Despite the promising findings, this study has important limitations. The relatively small sample size limits statistical power, particularly for detecting interaction effects; therefore, such results should be interpreted with caution. In addition, participants were assigned at the school level to minimise contamination, resulting in a cluster-based structure; however, multilevel modelling was not feasible due to the limited sample size, which may have influenced the precision of the estimates.

## Conclusion

The present study addressed the persistent research problem of attentional deficits in pupils with intellectual disability, a challenge that undermines learning and inclusion. While art therapy has traditionally been positioned as an enrichment activity, this study reconceptualized it as a structured cognitive intervention grounded in multisensory and neurodevelopmental principles. Little controlled evidence existed—particularly in Sub-Saharan Africa—regarding measurable impacts on attentional skills. By adapting a quasi-experimental design to the Nigerian educational context, the study bridged a major empirical gap and demonstrated the feasibility of culturally responsive art-based interventions.

The findings demonstrated three central results. First, art therapy significantly improved attention span compared to the control group, producing a large and educationally meaningful effect. Second, the degree of disability significantly predicted responsiveness, with pupils with mild intellectual disability achieving stronger outcomes than those with moderate disability. Third, the absence of a significant interaction effect indicated consistent benefits across severity levels, underscoring the adaptability and scalability of the approach. These outcomes confirm that structured art-based therapy can function as an evidence-based cognitive approach that enhances attention through sensory engagement, emotional regulation, and sustained focus.

The implications of this study are twofold. At a theoretical level, it strengthens understanding of the cognitive dimensions of art therapy by demonstrating measurable attentional gains within a Sub-Saharan African context. At a practical level, the results support integrating structured art-based therapy into special education curricula as a low-cost, scalable, and culturally adaptable method for improving attention and participation. Embedding art-based practices into teacher training programs could extend these benefits to inclusive classrooms in resource-constrained settings.

Future research should build upon these findings through larger, multi-site studies with longer follow-up periods to assess the durability of outcomes. Further exploration of mediating mechanisms, such as emotional regulation, motivation, and sensory integration, will clarify how art therapy produces its effects. Mixed-method and neurocognitive approaches may also help uncover underlying processes at behavioral and neural levels. Ultimately, this study establishes art therapy as a scalable, inclusive, and contextually adaptable intervention that can significantly contribute to educational equity and cognitive development among pupils with intellectual disability.

## Data Availability

The raw data supporting the conclusions of this article will be made available by the authors, without undue reservation.
